# Field studies of *Culex* mosquitoes in Tanzania and Kenya: A systematic review motivated by changing Rift Valley fever virus transmission patterns

**DOI:** 10.1111/mve.12811

**Published:** 2025-06-13

**Authors:** Catherine Andrews, Joshua Longbottom, Joel Lutomiah, Jennifer S. Lord

**Affiliations:** ^1^ Liverpool School of Tropical Medicine Liverpool UK; ^2^ Centre for Virus Research Kenya Medical Research Institute Nairobi Kenya; ^3^ Present address: UK Health Security Agency London UK

**Keywords:** arbovirus, *Culex*, Rift Valley fever virus

## Abstract

*Culex* mosquitoes are assumed to be secondary vectors of Rift Valley fever virus (RVFV), with *Aedes* being the most important for initiating outbreaks. However, environmental change may be affecting the role *Culex* species play in transmission. We aimed to curate a collection of published studies from Tanzania and Kenya, identify gaps in research concerning *Culex* communities and establish whether sufficient spatio‐temporal published data may be available for future meta‐analysis. This presents a first step in leveraging published data to better understand the role of *Culex* in maintaining RVFV transmission. We carried out a systematic search of the published literature using Web of Science for studies that sampled *Culex* in Tanzania or Kenya, up until the 28th April 2023. We determined motivations for studies, their duration and the geographic coverage in relation to an RVFV risk map. We then assessed species identification methods and how these may have impacted results. Of 275 studies, 17 explicitly stated the motivation for the study was RVFV. Despite most studies being motivated by other topics, there was good coverage of studies reporting mosquito sampling in areas associated with the risk of RVFV outbreaks. Fifty studies were at least 12 months in duration. In terms of species identification, studies were *c.* 14 times more likely to have identified more species than just the *Culex pipiens* complex if they stated that they used a *Culex* specific key. Although the majority of published studies sampling *Culex* in Kenya and/or Tanzania did not state RVFV explicitly as a key motivation for research, we propose that drawing on the data contained within these wider studies may still be of value for understanding how RVFV transmission is maintained. Our work here presents a first step to this end.

## Graphical abstract

- There were 275 studies reporting *Culex* abundance, 17 of which explicitly stated the motivation for the study was RVFV.
- Despite most studies being motivated by other topics, there was good coverage of studies reporting mosquito sampling in areas associated with the risk of RVFV outbreaks.
- Studies were *c.* 14 times more likely to have identified more species than just *Culex pipiens* complex if they stated that they used a *Culex*‐specific key.

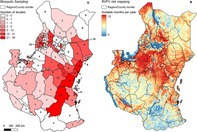

## INTRODUCTION

Rift Valley fever virus (RVFV), endemic across many areas of sub‐Saharan Africa, is a World Health Organisation priority pathogen, due to its outbreak potential and lack of a licensed human vaccine or other effective clinical countermeasures (World Health Organization, 2021). The virus is zoonotic and can be maintained between mosquitoes and ruminants including sheep, goats and cows, in addition to wildlife (Davies, 1975; Rostal et al., 2017; Wichgers Schreur et al., 2021). It can cause abortion in livestock and haemorrhagic fever in humans (World Health Organization, 2018). National epizootics of RVFV in Eastern and Southern Africa have historically been linked to drought followed by periods of heavy and persistent rainfall during El Niño‐Southern Oscillation events (Anyamba et al., 2021; Davies et al., 1985; Linthicum et al., 1999; Sumaye et al., 2013). Linthicum et al. (1985) showed that both naturally and artificially flooded dambos caused drought‐resistant *Aedes* eggs to hatch. They subsequently identified RVFV in emerging adult mosquitoes. From this research, the proposed cycle of RVFV before an epizootic in this context is therefore maintenance by transovarial transmission in *Aedes* populations. Continued inundation of dambos following prolonged rainfall allows hatching of large numbers of floodwater mosquito eggs resulting in an increase in floodwater *Aedes* population densities. These are then succeeded by large densities of *Culex* mosquito species in flooded areas. Floodwater species of *Aedes* mosquitoes that are infected serve to initiate mosquito‐host transmission, while *Culex* mosquitoes play a secondary role in transmitting virus from infected domestic animals to non‐infected animals and humans once this cycle has begun (Gibson et al., 2023; Linthicum et al., 1999).

Since the initial studies by Linthicum et al. (1985), the theory that *Aedes* are the primary vectors responsible for initiating outbreaks only during periods of high rainfall, followed by *Culex* amplification, has been widely held (for example as summarised in Mariner, Raizman, Pittiglio, Bebay, Kivaria, Lubroth, & Makonnen, 2022). In recent years, however, evidence of localised inter‐epizootic transmission of RVFV from mosquitoes to hosts has been reported, including seroconversion in both humans and livestock in Tanzania, Kenya, South Africa and Madagascar (Gray et al., 2015; Heinrich et al., 2012; Kumalija et al., 2021; LaBeaud et al., 2008, 2011; Muturi et al., 2023; Salekwa et al., 2019; Sumaye et al., 2013, 2015). Evidence has therefore accumulated in support of the hypothesis that RVFV can be maintained during inter‐epizootic periods at a relatively low level, or at a local scale, between hosts and mosquitoes (Lichoti et al., 2014; Muturi et al., 2023). This therefore calls into question the assumption that RVFV is maintained solely by vertical transmission in *Aedes* mosquitoes in between epizootics.

In addition to evidence of inter‐epizootic transmission, a change in RVFV geographic distribution during epizootics may also have occurred, albeit it is difficult to prove due to concurrent changes in awareness and reporting. For example, Sindato et al. (2014) reviewed RVFV epizootics in Tanzania between 1930 and 2007 and showed that more recent outbreaks involved a greater proportion of the country. While this should be interpreted with caution, it could be a consequence of increasing livestock densities and changing vector larval habitats, for example through expansion of crop cultivation, over the past few decades. According to FAOSTAT (FAO, 2022), in the 1960s in Tanzania there were *c*. 8 million cattle, and this had risen to *c*. 31 million in 2021. Similarly, rice, which can provide suitable larval habitats for mosquitoes, covered *c*. 80,000 ha in the 1960s and this had risen to >1.3 million ha by 2021. In areas where irrigation systems and livestock are present, this may lead to an increase in the mosquito to host ratio (Sang et al., 2017). Changes in livestock systems, land use and the environment have possibly contributed to changes in RVFV transmission patterns and will likely continue to do so (Gibson et al., 2023).

RVFV can infect multiple host species and many mosquito species across *Aedes*, *Culex*, *Mansonia* and *Anopheles* genera (Kroeker et al., 2020; Sang et al., 2010; Turell et al., 2008). The relative role of any mosquito species in transmission depends on their ability to become infected and subsequently transmit the virus, their host feeding behaviour, and their relative abundance. Vector competence experiments within a laboratory setting have demonstrated that *Culex antennatus*, *Culex zombaensis*, *Culex poicilipes*, *Culex quinquefasciatus* and *Culex pipiens* can develop a disseminated infection and subsequently transmit RVFV (Brustolin et al., 2017; Ndiaye et al., 2016; Turell et al., 2007, 2008) and *Culex* have been suggested as primary vectors in some outbreaks. For example, in Egypt, *Cx. antennatus*, which breeds in rice fields and irrigation ditches, was implicated as the primary vector during an outbreak of RVFV in humans, cattle and sheep in 2003, and *Cx. pipiens* was implicated in an outbreak in 1977 (Hanafi et al., 2011). During an outbreak in Rwanda in 2022, *Cx. quinquefasciatus* was the most common species collected, and a single pool of this species tested positive for RVFV before the outbreak and two pools during the outbreak (Nsengimana et al., 2025). Despite the competence of several *Culex* species for RVFV and established importance for secondary/onward transmission during epizootics, to our knowledge, there is a gap in understanding the role *Culex* may play in maintaining focal transmission between hosts in between RVFV national epizootics.

As vector abundance is a key parameter in determining the relative role of a mosquito species in transmission, a review of findings from existing field studies for *Culex* may contribute insight. Here, we focus on studies that reported sampling *Culex* mosquitoes in Tanzania and Kenya; two countries where both national epizootics as well as focal inter‐epizootic transmission have occurred. Our aim was to curate a collection of published studies from Tanzania and Kenya, identify gaps in research concerning *Culex* communities and establish whether sufficient spatio‐temporal published data may be available for future meta‐analysis. To this end, we aimed to carry out a systematic review of the literature to determine: (i) what studies have been carried out that involve sampling *Culex* in Tanzania and Kenya; (ii) the extent to which longitudinal sampling has taken place to enable characterisation of *Culex* population dynamics; (iii) the geographic coverage of studies; (iv) whether sampling has been carried out in areas most at risk of RVF outbreaks; and (v) how many studies use morphological keys or molecular approaches to identify *Culex* mosquitoes to species and the potential consequences for identification. We acknowledge that the work presented here is just a first step in potentially leveraging published data to better understand the role of *Culex* in maintaining focal RVFV transmission.

## METHODS

### Systematic search of the literature

Our systematic review was conducted following the Preferred Reporting Items for Systematic Reviews and Meta‐Analysis (PRISMA) guidelines where possible (Files S2 and S3). The review was not registered before data extraction took place. The initial identification of search terms and combinations were carried out by C.A. and J.L. to ensure that a complete set of studies were collected. Searches were made initially on 24th March 2023 on all Web of Science's databases (1950s–present), collated by C.A. and checked by J.L. during the decision‐making process through in‐person discussions. The search was updated on 28th April 2023. The search terms included ‘*Culex* AND Tanzania’ OR ‘*Culex* AND Kenya’, with these search terms used under both the Topic and Abstract categories.

Citations were exported into Endnote reference manager (Endnote 20) and Microsoft Excel. C.A. manually and automatically searched the titles and abstracts of the references for studies that had carried out field collections of mosquitoes in Tanzania or Kenya. Any duplicate citations were removed. Automatic searches used Microsoft Excel's IF function to search for ‘*Culex*’ and/or ‘*Cx*’ and ‘Tanzania’ and/or ‘Kenya’ in the title and abstract. If both terms were present, the study was included for full‐text review. If neither, or only one of these search terms were found, C.A. manually read the title and abstract to assess the eligibility. Exclusion criteria at this stage of the review included the study not involving mosquitoes, the study not being in Tanzania or Kenya, or no mosquito sampling occurring. These were reviewed by J.L. to confirm that agreements were made on these initial inclusions/exclusions. Once these were agreed between contributors, full texts were retrieved.

We read the full texts of articles included at the title/abstract stage and excluded studies that did not report any *Culex* or reported mosquitoes as culicine only, rather than separating by genus. We also excluded studies that did not include any entomological field sampling, including studies that used Tanzanian or Kenyan *Culex* strains in laboratory testing. Review papers, meta‐analyses of multiple countries, conference abstracts or posters, books, opinions and duplicate records were also excluded. Conference proceedings were not included as they generally did not contain sufficient information with respect to study details and results.

### Data extraction

All extracted information from full texts was entered into a Microsoft Excel spreadsheet. A single article was written in French and was translated to English, using the image translate feature in Google Translate. Variables extracted during the full‐text screening are explained in Table 1.

**TABLE 1:** Details of the data extracted from published articles reporting results of *Culex* field sampling.

Variable name	Description of the objective
Publication year	Year of publication
Country	The country (TZA/KEN/Both) where the entomological sampling took place
Village/ward	The village or ward where the sampling took place
District/sub‐county	The district (TZA) or sub‐county (KEN) in which sampling took place
Region/county	The region (TZA) or county (KEN) in which the sampling took place
Trapping method	The trapping method used to sample the mosquitoes, for example, CDC light trap, BG sentinel
Specimen caught	The life stage of mosquito caught via the sampling approach, for example, eggs, larvae, pupae or adults
Specimen ID	The life stage of mosquito used for genus/species identification, for example, eggs, larvae, pupae or adults
ID method	Which identification technique was used to identify the mosquito genus and/or species. This was a fixed entry field detailing either: ‘morphology’ or ‘molecular techniques’ encompassing approaches such as PCR, sequencing or a combination of both
Morphological keys	The morphological key that was used for identification, if stated
Molecular marker used	The molecular marker, if used, and whether it was only used on *Anopheles* specimens
TZA/KEN *Culex* mosquito strain	Yes or no, for studies that also had a laboratory component, concerning whether the study used a *Culex* strain originating from TZA or KEN
*Culex* genus only	Whether mosquitoes were identified to genus level only
Species	List of the *Culex* species identified in the study. Members of the *Culex pipiens* complex, including *Culex quinquefasciatus*, were recorded as *quinquefasciatus*/*pipiens*
Trap composition	The percentage of the total mosquito catch that were identified as *Culex* mosquitoes, to the nearest whole number
Objective	The objective(s) for the study
Date	Dates the mosquito sampling took place over

Abbreviations: BG, Biogents; CDC, Centre for Disease Control and Prevention; KEN, Kenya; PCR, polymerase chain reaction; TZA, Tanzania.

The extraction of information was carried out only by C.A. for 90% of studies, but 5% of studies were randomly assigned to J.L. and 5% to J.S.L. to be double‐checked. Any discrepancies found between these extractions were resolved through in‐person discussions. For several studies, locations were restricted to village names or historic province/administrative names. Since 2010, Kenya introduced geographical units that replaced the provincial system (Kenya Law Reports, 2010). Similarly, for Tanzania, additional administrative regions have been created as recently as 2016, so more up‐to‐date data extraction of localities was used (Mwakyusa, 2016). The updated county/region and district/sub‐county for a given location was determined using OpenStreetMap© (OpenStreetMap©, 2024). The motivation for each study was identified by reading the aims, usually at the end of the introduction section, and placing into at least one of the following categories: (1) RVFV; (2) malaria; (3) arboviruses; (4) general mosquito ecology; (5) trapping method comparisons; (6) helminth infections; (7) irrigation/rice; (8) immature stage ecology; and (9) mosquito control including insecticides, attractants and repellents.

### Data analysis

We totalled numbers of included published papers by publication decade to show temporal trends in the publication of studies reporting *Culex* sampling. We summarised studies by key theme, duration of sampling and geographical coverage, by country, region and district for Tanzania and by counties and sub‐counties for Kenya. Duration of sampling was estimated using, where given, study start and end dates, but we did not determine whether this was seasonal or continuous sampling for the study period. With respect to geographical coverage, we then combined our extracted data with information on RVFV risk provided by Hardcastle et al. (2020). We summarised the number of studies that carried out *Culex* sampling at the region/county level and at the district/sub‐county level. The number of published studies by region/county/district/sub‐county was added to the attribute table of administrative boundary shapefiles from The Humanitarian Data Exchange so that they could be viewed next to the RVFV risk map (Exchange HD, 2018; Exchange HD, 2023).

Next, we looked at *Culex* species identification. We summed the total number of *Culex* species reported across all studies and the number of studies reporting *Culex* identification to species level. Using the studies that identified *Culex* down to species level, studies were grouped according to the identification methods that they reported to have used to identify to species. There were seven distinct groups: (1) morphology only with a *Culex* key; (2) morphology only without a *Culex* key; (3) morphology only with no key stated; (4) morphological identification with a *Culex* key and molecular confirmation; (5) morphological identification without a *Culex* key and molecular confirmation; (6) morphological identification and molecular confirmation with no key stated; and (7) no identification method stated. For each of these groups, the mean number of *Culex* species identified, reported to the nearest integer, was calculated as well as the proportion of studies that identified more than just *Cx. pipiens* complex. We calculated the odds of a study identifying more *Culex* species than just the *Cx. pipiens* complex if the use of a specific *Culex*‐specific taxonomic key was reported in the methods, as compared with either no mention of a key or only keys for *Anopheles* or *Aedes*. When calculating the odds, we excluded nine studies that used molecular techniques so that we were only comparing studies based on the approach to morphological identification.

## RESULTS

### Literature search summary

**FIGURE 1:**
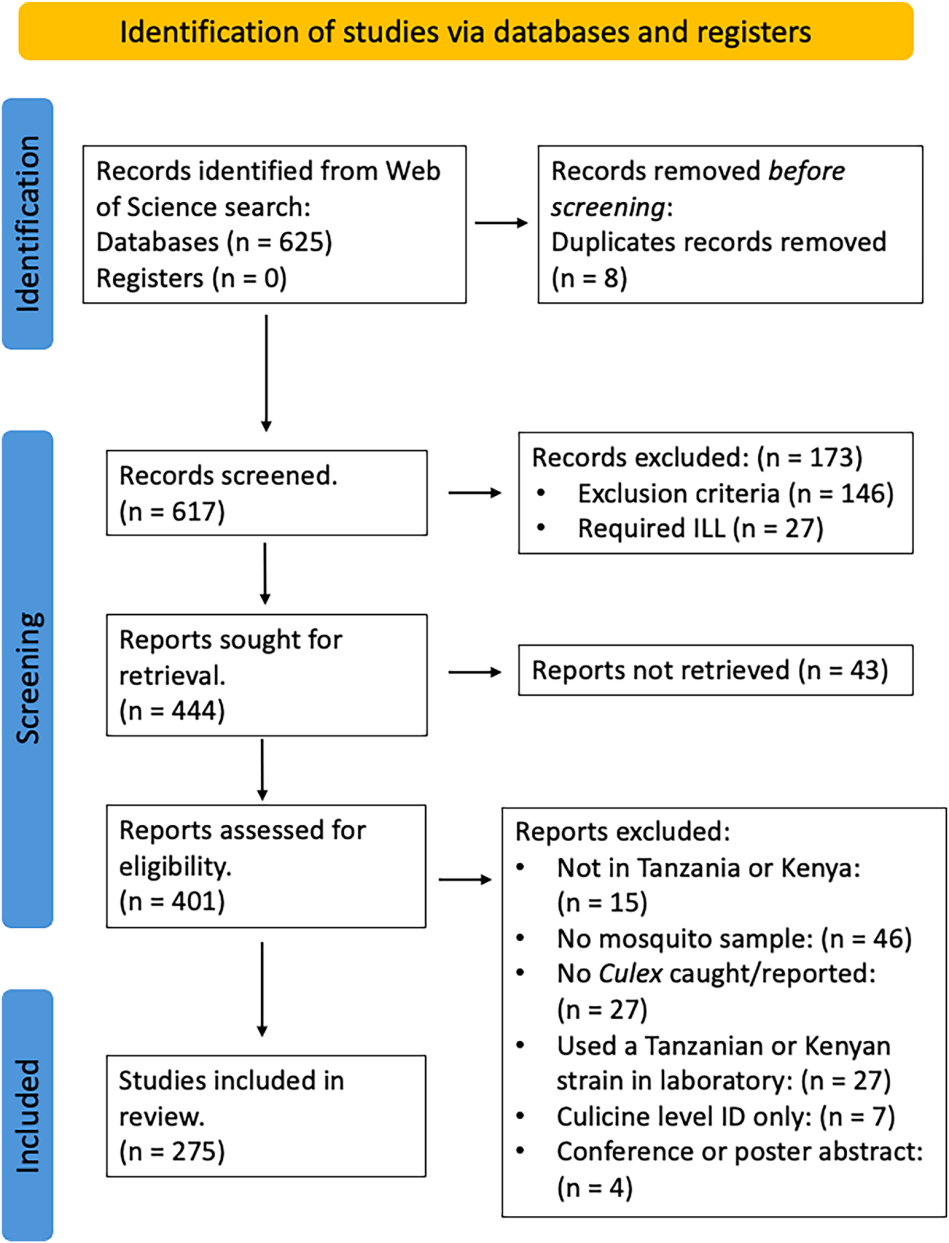
Preferred Reporting Items for Systematic Reviews and Meta‐Analyses (PRISMA) flow diagram of the search, screening and inclusion process of studies reporting *Culex* collections in Tanzania or Kenya.

**FIGURE 2:**
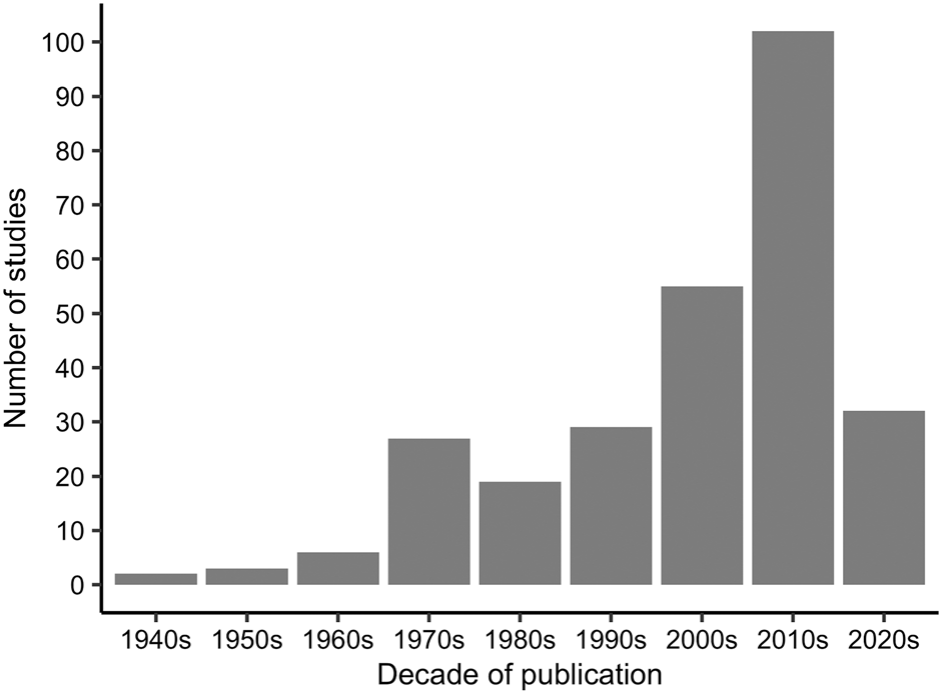
Number of published studies reporting *Culex* sampling in Tanzania or Kenya by decade.

We retrieved 625 references from the Web of Science. After removal of duplicates (*n* = 8), 617 paper titles and abstracts were screened. There were 27 citations for which we could not find abstracts. After screening, 444 articles were included for full‐text eligibility assessment and, of these, the full text could not be obtained for 43 studies, and 173 were excluded as they did not meet the inclusion criteria outlined in the methods. This left 275 papers which we identified as eligible for inclusion (Figure 1). File S1 contains the complete reference list of all studies included in our review (Files S2 and S3 contain PRISMA 2020 checklists). Of these studies, 69% (189/275) were published after 2000 (Figure 2).

### Key themes and duration of sampling

**FIGURE 3:**
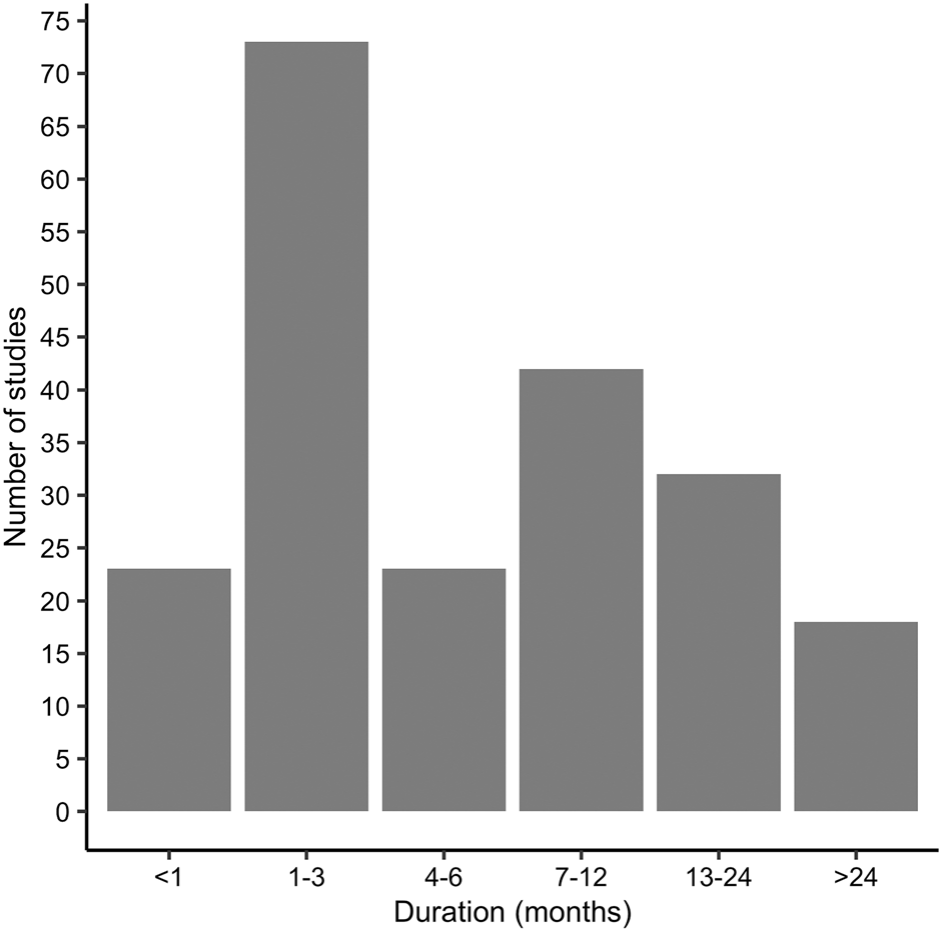
Number of published studies reporting *Culex* sampling by duration of study.

RVFV was stated as a key motivation in 6% (17/275) of studies. Other key themes of studies included general mosquito ecology (28%, 77/275), malaria (20%, 54/275), arboviruses (17%, 47/275), trapping method comparisons (17%, 48/275), helminth infections (12%, 33/275), studies sampling in irrigated areas (6%, 17/275), immature stage ecology (6%, 17/275) and mosquito control including insecticides (14%, 39/275), attractants (6%, 17/275) and repellents (2%, 6/275). Approximately half of the studies (54%, 115/211), where dates were given, were less than 6 months in duration, while 50 studies (24%) included sampling spanning >12 months (Figure 3).

### Geographical coverage

**FIGURE 4:**
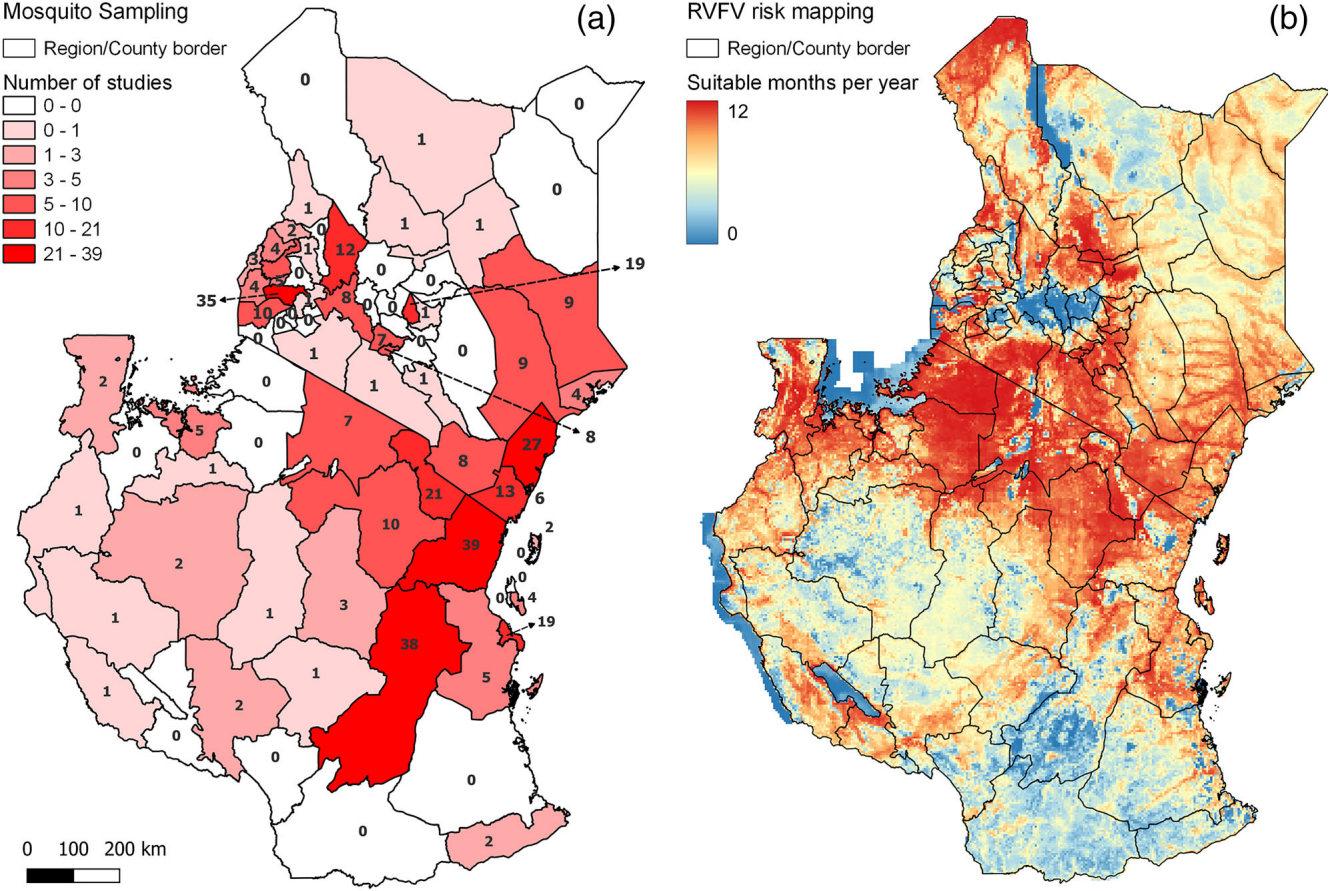
Comparison of risk of Rift Valley fever (RVF) risk and number of studies reporting *Culex* sampling across Tanzania and Kenya. (a) Number of studies reporting *Culex* sampling. (b) 5 × 5‐km pixel‐level risk map from Hardcastle et al (Hardcastle et al., 2020) of the average number of months suitable for RVF virus (RVFV) based on environmental suitability predictions from years between 1995 and 2016.

Half of the studies (138/275) reported mosquito collections in Kenya, 47% (129/275) were carried out in Tanzania and 3% (8/275) of studies collected mosquitoes in both countries. With respect to identifying study locations within the country, there were just four studies that did not report a region or county and seven that did not report a district or sub‐county. From the remaining studies, we found that 68% (21/31) of the regions in Tanzania have been subject to at least one field study involving *Culex* collections between November 1942 and March 2022 (Figure 4, Appendix S1). The regions that have been most frequently sampled include Tanga (28%, 39/137) and Morogoro (28%, 38/137), followed by Kilimanjaro (15%, 21/137) and Dar es Salaam (14%, 19/137). At a smaller administrative level, we found that there have been entomological collections in 33% of the 170 districts of Tanzania (Appendix S1). Districts in Tanzania where the majority of *Culex* sampling has taken place include Muheza within the Tanga region (19%, 27/137), Kilombero (15%, 21/137) and Ulanga (14%, 19/137) in the Morogoro region and Moshi (12%, 17/137) in the Kilimanjaro region.

In Kenya, *Culex* sampling has taken place in 64% of the 47 counties (Figure 4). Counties with the highest frequency of studies include Kisumu (24%, 35/146), Kilifi (18%, 27/146) and Kirinyaga (13%, 19/146). At the smaller scale in Kenya, 28% of 290 sub‐counties have been subject to *Culex* sampling. Kenyan sub‐counties where *Culex* sampling has most frequently taken place include Nyando (17%, 25/146) of Kisumu County, followed by Mwea (12%, 17/146) of Kirinyaga County and Baringo South (10%, 15/146) of Baringo County (Appendix S1).

With respect to RVFV risk, as estimated by Hardcastle et al. (2020) and *Culex* sampling coverage, we observed that there were between seven and 39 studies in each region/county across the south of Kenya and northwest of Tanzania which were considered as high risk for RVFV for most of the year. However, there were some areas that were determined as high risk and have not been subject to sampling, including Mara and Simiyu regions of Tanzania (Figure 4, Appendix S1).

### Culex *species identification*

A total of 61 different *Culex* species have been reported across Tanzania and Kenya in the reviewed studies. Of the included studies, 77% (212/275) reported identification of *Culex* mosquitoes to species level. Of these studies, most (95%, 202/212) reported use of only morphological techniques for identification or did not explicitly state the method of identification used. A single study (Jones et al., 2012) was excluded from further summaries as the authors appear to have carried out only molecular identification on the *Culex* samples. For those studies that reported *Culex* to species level, 38% (80/211) explicitly reported the use of at least one *Culex*‐specific key. The *Culex*‐specific keys used in studies were Edwards (Edwards, 1941), Hopkins (Hopkins, 1952), Jupp (Jupp, 1971), Mattingly (Mattingly, 1971), Gillet (Gillet, 1972), Darsie & Ward (Darsie & Ward, 1981), Highton (Highton, 1983), Harbach (Harbach, 1988), Jupp (Jupp, 1996), Snell (Snell, 2005), Azari‐Hamidian & Harbach (Azari‐Hamidian & Harbach, 2009), Walter Reed Biosystematics Unit (Walter Reed Biosystematics Unit, 2020) and a CDC key (Center for Disease Control and Prevention, 2006) (File S1). The remaining studies reported use of either *Anopheles* or *Aedes* keys or did not state the key that they used.

**FIGURE 5:**
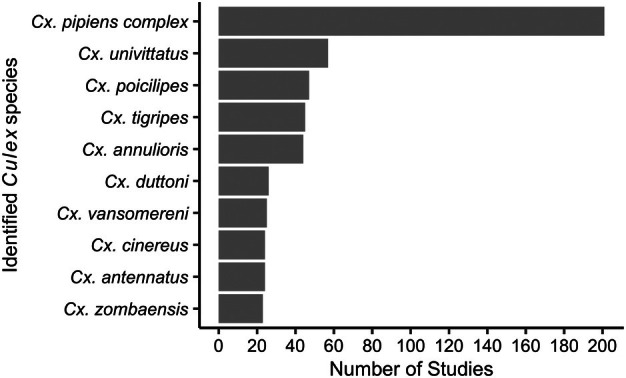
Number of studies reporting the occurrence of each of the top 10 most frequently identified *Culex*. The members of *Culex pipiens* complex are treated as one species group.

*Culex pipiens* complex was most frequently reported (95% of studies, 201/211), followed by *Culex univittatus* (27% of studies, 57/211), *Cx. poicilipes* (22% of studies, 47/211) and *Culex tigripes* (21% of studies, 45/211) (Figure 5). Ajamma et al. (2016) reported the greatest species richness with 24 *Culex* species identified across two Kenyan counties: Baringo and Homa Bay, using both morphology and molecular techniques with the use of a *Culex* specific key. Following this was Linthicum et al. (1985), using five morphological keys including those that were *Culex* specific. This study identified 23 different *Culex* species across five counties in Kenya.

There were 32 studies that reported the use of molecular techniques for mosquito identification; most (72%, 23/32) were for *Anopheles* only and >50% were malaria focused (12/23). In comparison, only nine studies (publication years 2012–2023) used molecular techniques on *Culex* specimens to provide further insight into species‐level identification. Five of these studies used amplification of cytochrome c oxidase subunit 1 (COI) alone or in combination with the internal transcribed spacer region (ITS) 2 and DNA barcoding. Three relied on polymerase chain reaction (PCR) using the acetylcholinesterase‐2 (ACE2) locus to distinguish *Cx. quinquefasciatus* from the *Cx. pipiens* complex (Iwashita et al., 2018; Matowo et al., 2019; Silva Martins et al., 2019). One study (Osei‐Poku et al., 2012) relied on sequencing, using ITS1, for *Culex* species identification using DNA barcoding, and a single study sequenced ‘representative mosquitoes’ using COI, with the remaining being morphologically identified (Musa et al., 2020). For the nine studies that used both morphological and molecular approaches for *Culex*, an average of 7.7 *Culex* species were identified, whereas those that only used morphological techniques identified on average 3.2 species. Studies with no reported identification method had the lowest mean *Culex* species identified of 1.8. Of those using morphology and molecular methods, 67% (6/9) used a *Culex* specific key during morphological identification of the mosquitoes (File S1).

**FIGURE 6:**
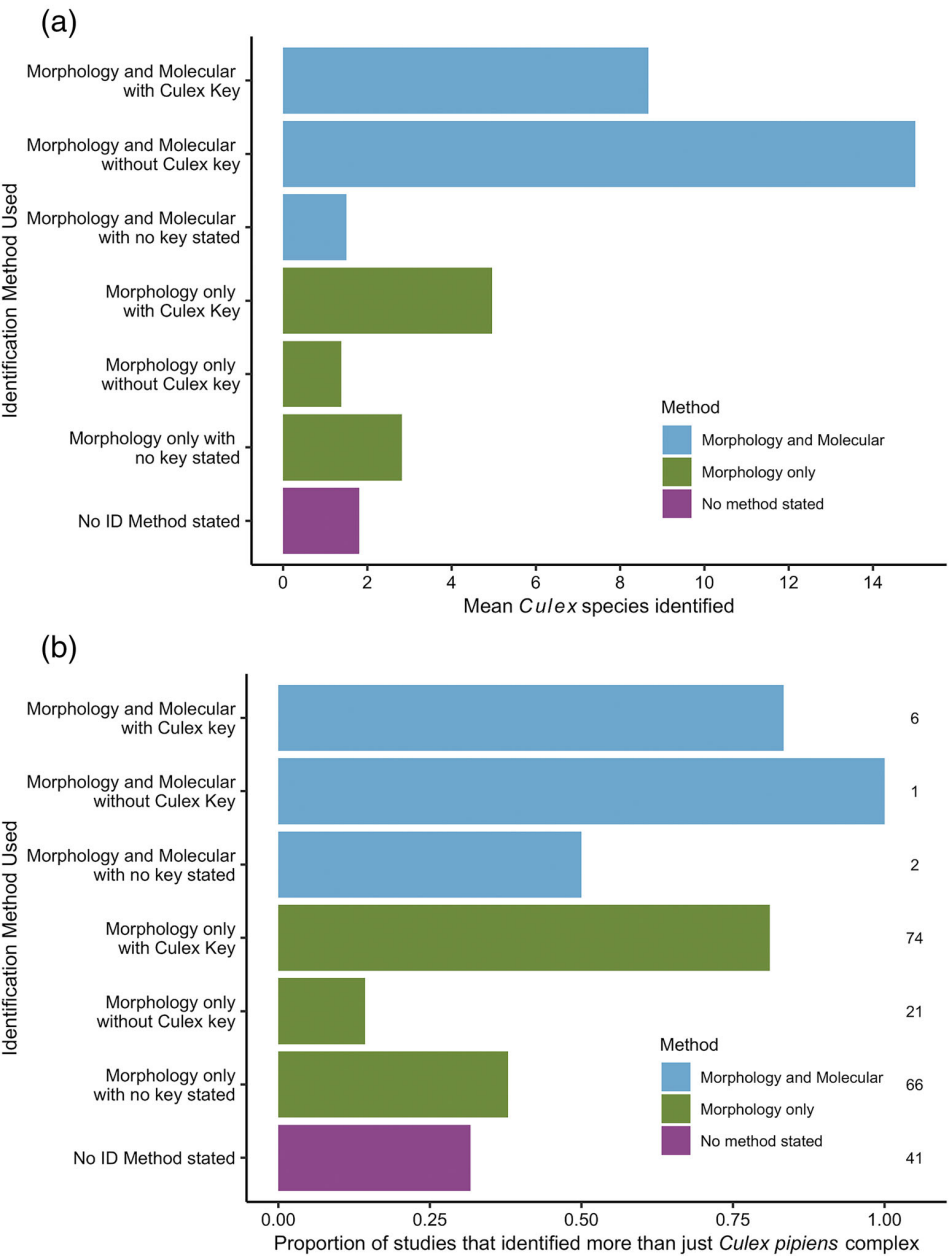
Association of the species identification method(s) used with *Culex* species identification results. (a) Mean number of *Culex* species identified by studies using different combinations of molecular and morphological approaches. (b) Proportion of studies reporting *Culex* species other than just *Culex pipiens*/*quinquefasciatus* according to identification method(s) used.

Collectively, the group of studies with both the lowest mean *Culex* species identified and proportion of studies that identified more than just *Cx. pipiens* complex were the studies using morphological techniques alone without the use of a *Culex* specific key (10% of the studies that identified mosquitoes to species level, 21/211) (Figure 6a). The group that had a greater proportion of studies reporting more than just *Cx. pipiens* complex were those studies that used both morphology and molecular techniques with a *Culex* specific key (3% of studies, 6/211). Studies using molecular methods tended to identify more species on average, and we found that studies were *c*. 14 times more likely to have identified more species than just those in the *Cx. pipiens* complex if they stated that they used a *Culex* specific key (Figure 6b).

Of the 55 studies that were malaria focused, 45% reported *Culex* mosquitoes to species level, but only 16% (4/25) employed a *Culex*‐specific morphological key (File S1). However, of these, 76% (19/25) reported *Cx. pipiens* complex only. Molecular methods were used in 64% (35/55) of the malaria‐focused studies, although this was only carried out on *Anopheles* samples. The average number of *Culex* species identified in malaria studies was 0.6, compared to 3.1 for studies with other reasoning.

## DISCUSSION

Although the majority of published studies sampling *Culex* in Kenya and/or Tanzania did not state RVFV epidemiology explicitly as a key motivation for research, we propose that drawing on the data contained within these wider studies may still be of value for understanding how RVFV transmission is maintained in mosquitoes and hosts in between national epizootics. In terms of temporal information, there were 50 studies that were at least 12 months in duration, which may contain useful data to inform models of potential vector population dynamics. With respect to spatial information, the studies provided good geographical coverage of both countries, and there was a relatively high number of studies from areas where there have recently been outbreaks, including Garissa and Baringo Counties in Kenya and Arusha District in Tanzania.

A high frequency of studies sampling mosquitoes in certain areas, including Mwea in Kenya and Moshi in Tanzania, can be explained by the presence of large irrigation schemes, the Mwea Irrigation Scheme and Lower Moshi Irrigation Scheme, respectively. These irrigation schemes provide larval habitats and subsequent high mosquito population densities, relative to other areas, potentially increasing the risk of both malaria and RVFV. Additionally, districts like Muheza in Tanzania, where sampling was high, are the locations of vector control trial sites.

Although many of the studies we reviewed were not focussed specifically on *Culex* mosquitoes, we note that *Culex* was often the most dominant genus caught, and their role as vectors maintaining RVFV in between national epizootics should be considered. The FAO recommends mosquito surveillance as part of effective RVF control based on a One Health approach, and *Culex* catch data from the published studies we have gathered could prove useful towards this by better understanding potential vector population dynamics in at risk areas (Mariner, Raizman, Pittiglio, Bebay, Kivaria, Lubroth, et al., 2022).

A total of 61 different *Culex* species across Tanzania and Kenya have been recorded in the published studies we reviewed. Of these, several species have already been implicated in RVFV transmission in Kenya or elsewhere. For example, *Cx. univittatus*, *Cx. quinquefasciatus*, *Cx. poicilipes* and *Culex bitaeniorhynchus* collected during the 2006–2007 RVF outbreak in Kenya were RVFV positive (Sang et al., 2010). While this does not confirm that they were involved in transmission, many *Culex* species have been found to become infected and subsequently transmit RVFV under experimental conditions (Amraoui et al., 2012; Bergren et al., 2021; Ndiaye et al., 2016; Talavera et al., 2018; Turell et al., 2007). *Culex antennatus* was implicated by Hanafi et al (Hanafi et al., 2011) as a major vector during an RVF outbreak in Egypt due to its catch abundance (95.8% of mosquitoes caught) and three RVFV positive pools. RVFV was isolated in *Culex tritaeniorhynchus* pools in Saudi Arabia during the 2000 epidemic (Jupp et al., 2002). *Culex zombaensis*, identified in 23 studies, has had virus isolated from it during both the South African outbreak of 1981 and during a livestock outbreak in Kenya (Logan et al., 1991; McIntosh et al., 1983). A better understanding of the ecological niche of the most common species, their population dynamics in Kenya and Tanzania, and their competency for RVFV would inform our understanding of RVFV transmission foci and the role of *Culex* in maintaining inter‐epizootic transmission.

One potential barrier to using the data within these studies to better understand *Culex* species ecology and their potential contribution to RVFV transmission, however, is the validity of species level identification, especially of species complexes. Most studies assessed here did not use molecular analyses to confirm the morphological identification of complexes such as *Cx. pipiens* complex, comprising *Cx. pipiens pipiens* and *Cx. pipiens quinquefasciatus*. There was evidence that studies were more likely to report fewer species, or only *Cx. pipiens* spp., when only morphological identification was carried out without the use of a *Culex* specific key. *Cx. quinquefasciatus* was the most frequently identified *Culex* species, 146 times more than the next most frequent species, *Cx. univittatus*. We suggest that it is possible that some studies that only reported *Cx. quinquefasciatus* may have mis‐identified other species. In addition, Mutebi et al. (2012) noted conflicting species descriptions of *Culex neavei* collected in Uganda between available *Culex* keys. This highlights the difficulty in the morphological identification of some *Culex* species and the need for the continued investment in accomplished taxonomists.

Scott et al. (1993) developed a PCR that separates five members of the *Anopheles gambiae* complex based on the differences in the nucleotide sequence of the ribosomal DNA intergenic spacers, capable of differentiating between species and interspecies hybrids. Additionally, a similar method is available for members of the *Anopheles funestus* group (Koekemoer et al., 2002). These techniques also exist for species of the *Cx. pipiens* complex, their hybrids and sibling species (Smith & Fonseca, 2004). Separation of species relies on polymorphisms within the acetylcholinesterase‐2 (ACE2) locus and differences in band size (Smith & Fonseca, 2004). Further molecular approaches could be developed in a similar way for the other common *Culex*. While we would not expect the use of routine molecular analysis to determine mosquito species, molecular analyses could be useful for researchers new to *Culex* taxonomy, or when studying mosquito communities in new geographic areas, to corroborate results from morphology. Capacity strengthening for *Culex* taxonomy should be considered as part of any RVFV research and surveillance initiatives.

Our study only focuses on published literature‐based studies. However, we are aware of the potential for additional sources of data that we may have missed, which could complement the data in the published studies. We acknowledge that 90% of the paper screening and data extraction was carried out by a single person and that this may have introduced a small amount of human error. However, 10% of articles were cross‐checked by other co‐authors to ensure that there was at least general agreement concerning how each aspect of the data extraction was done. We have provided the raw extracted results as a supplementary file and hope that by having a collated list of existing studies reporting *Culex* abundance, these could be further interrogated for useful data. Indeed, our next steps where possible will be to extract information detailing the count of mosquitoes per trap from these studies, at least at the genus level, to inform spatio‐temporal analyses of *Culex* abundance.

There is now strong evidence for the maintenance of RVFV in mosquitoes and vertebrate hosts on a regional or local scale (between villages), in between outbreaks that are recognised as national epizootics (Gray et al., 2015; Heinrich et al., 2012; Kumalija et al., 2021; LaBeaud et al., 2008, 2011; Muturi et al., 2023; Salekwa et al., 2019; Sumaye et al., 2013, 2015). This has consequences for control planning because the force of infection on a local scale, how this varies spatially, and the drivers of that variation will affect the risk of a national epizootic through the relative proportions of susceptible and recovered/immune livestock at any given place and time. The FAO Action Framework currently advises preventive control strategies around inter‐epizootic, pre‐epizootic, epizootic and post‐epizootic periods. Targeted vaccination and vector control at risk hotspots are proposed as part of pre‐emptive interventions when a risk alert for RVFV is raised (Mariner, Raizman, Pittiglio, Bebay, Kivaria, Lubroth, et al., 2022). We argue that there is a need to better understand where inter‐epizootic transmission is occurring on a local scale to inform this. In turn, identifying drivers of localised transmission between hosts and vectors requires determination of potential vector community composition and species population dynamics. We hope that the information we have collated here will be a useful step towards making the most of published studies to that end.

## AUTHOR CONTRIBUTIONS

**Catherine Andrews:** Investigation; writing – original draft; methodology; visualization; writing – review and editing; formal analysis; validation. **Joshua Longbottom:** Visualization; writing – review and editing; conceptualization; validation. **Joel Lutomiah:** Writing – review and editing; visualization. **Jennifer S. Lord:** Writing – original draft; writing – review and editing; conceptualization; investigation; methodology; validation; visualization; formal analysis; supervision.

## CONFLICT OF INTEREST STATEMENT

The authors declare no conflicts of interest.

## Supporting information


**Data S1.** Structured reflexivity statements.

**Appendix S1.** Supplementary mapping and risk figures.

**File S1.** Extracted data used in analysis, *Culex* key information and full reference list.

**File S2.** PRISMA 2020 abstract checklist.

**File S3.** PRISMA 2020 expanded checklist.

## Data Availability

The data that supports the findings of this study are available in the supplementary material of this article.
