# Supplementary material for: Field studies of Culex mosquitoes in Tanzania and Kenya: A systematic review motivated by changing Rift Valley fever virus transmission patterns
Source: Med Vet Entomol. 2025 Jun 13;39(4):689–700. doi: 10.1111/mve.12811 (PMC12586299; doi:10.1111/mve.12811)
Supplement: Supplementary file 1 — Data S1. Structured reflexivity statements. [file MVE-39-689-s002.docx]

Please complete all sections relevant to your research study

| Study conceptualisation | |  |
| --- | --- | --- |
| Please justify the choice to conduct the study in and/or acquire samples from the LMIC location | | The work involved systematic review of published studies carried out in Tanzania and Kenya with a focus on Rift Valley fever virus. We had a technician based at LSTM funded by a NERC project on RVFV, who had some spare time to do this review. Although the wider funded project was in collaboration with partners at the Tanzanian Veterinary Laboratories Agency, this piece of work was carried out with our co-author Joel Lutomiah (J.L.) who is based at KEMRI, Kenya, given his expertise in mosquitoes. |
|  | How does this study address local research priorities and how were local researchers involved in study design? | Once Catherine Andrews had pulled the publications together, we discussed the data extracted with J.L. and he worked with us on the results and discussion. RVFV is a local research priority in both Tanzania and Kenya. |
| Research management | |  |
|  | How has funding been used to support the local research team(s)? | There was no funding for this review *per se* – aside from the salary of the LSTM-based technician. It was essentially a ‘side-project’. |
| Data acquisition and analysis | |  |
|  | How are research staff who conducted data collection acknowledged? | NA |
|  | How have members of the research partnership been provided with access to study data and analytical tools? | NA |
|  | If genetic resources were shared, how were the principles of the Nagoya Protocol on the equitable sharing of benefits | NA |
| Data interpretation | |  |
|  | How have research partners collaborated in interpreting study data? | We collectively wrote the results and discussion. |
| Drafting and revising for intellectual content | |  |
|  | How were research partners supported to develop writing skills? | We supported each other; learning collectively via tracked-changes in drafts of the manuscript. |
| Authorship | |  |
|  | How is the leadership, contribution and ownership of this work by LMIC researchers recognised within the authorship? | Joel Lutomiah provided advice and input concerning the interpretation of the results. |
|  | How have early career researchers across the partnership been included within the authorship team? | Catherine Andrews was a technician at time of writing and was given this as something she could lead on, acknowledging that this is often something that technician’s do not get the opportunity to do. |
|  | How has gender balance been addressed within the authorship? | This was 50/50. |
| Training | |  |
|  | How has the project contributed to training of LMIC researchers? | NA |

Adapted from: Morton et al. 2021 “Consensus statement on measures to promote equitable authorship in the publication of research from international partnerships” <https://doi.org/10.1111/anae.15597>
