# Supplementary material for: Field studies of Culex mosquitoes in Tanzania and Kenya: A systematic review motivated by changing Rift Valley fever virus transmission patterns
Source: Med Vet Entomol. 2025 Jun 13;39(4):689–700. doi: 10.1111/mve.12811 (PMC12586299; doi:10.1111/mve.12811)
Supplement: Supplementary file 2 — Appendix S1. Supplementary mapping and risk figures. [file MVE-39-689-s004.docx]

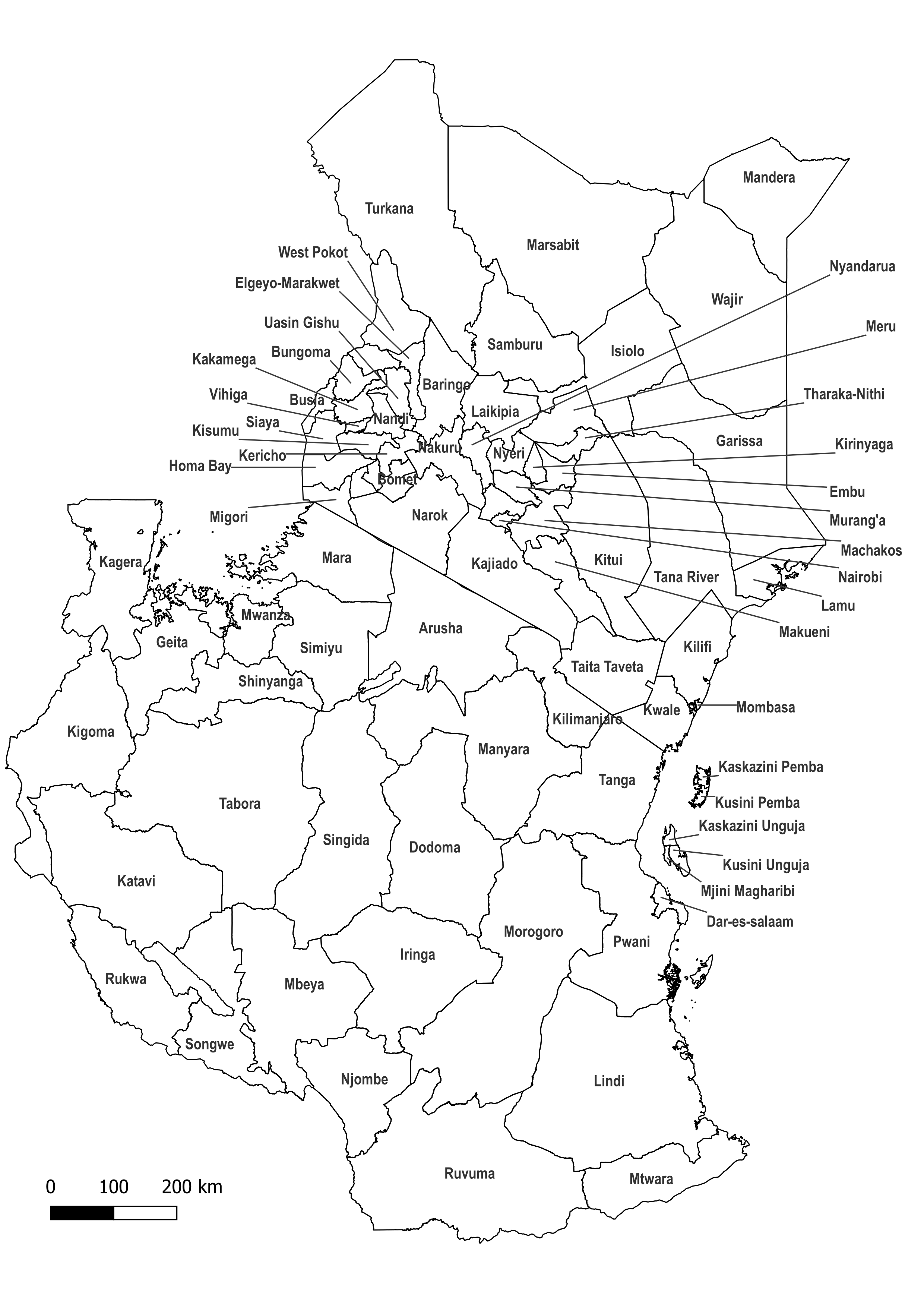


**S1 Figure. Labelled map of Kenya and Tanzania at the region/county level.**


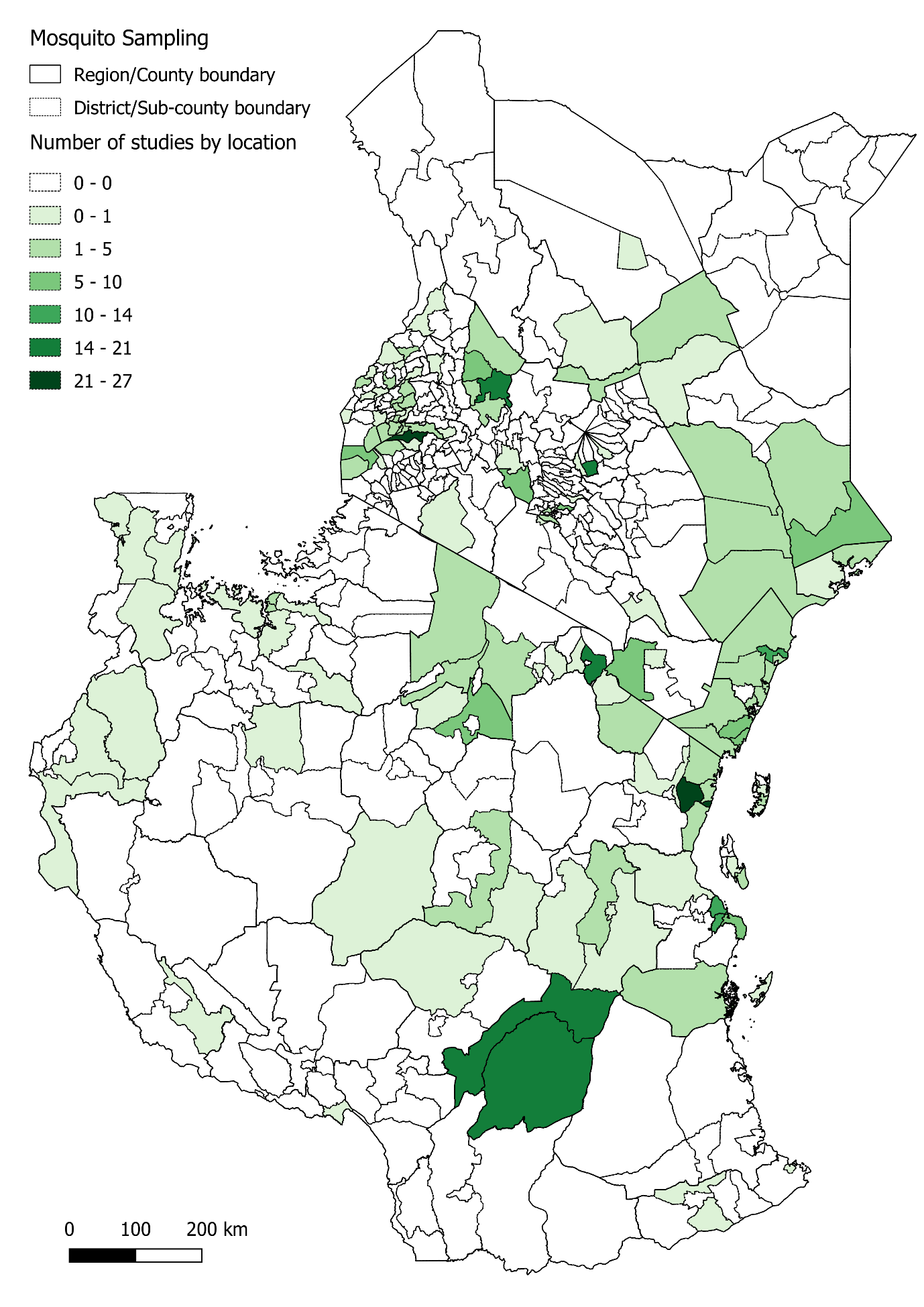


**S2 Figure. Count data of mosquito sampling in Kenya and Tanzania at the Sub-county/District level.**


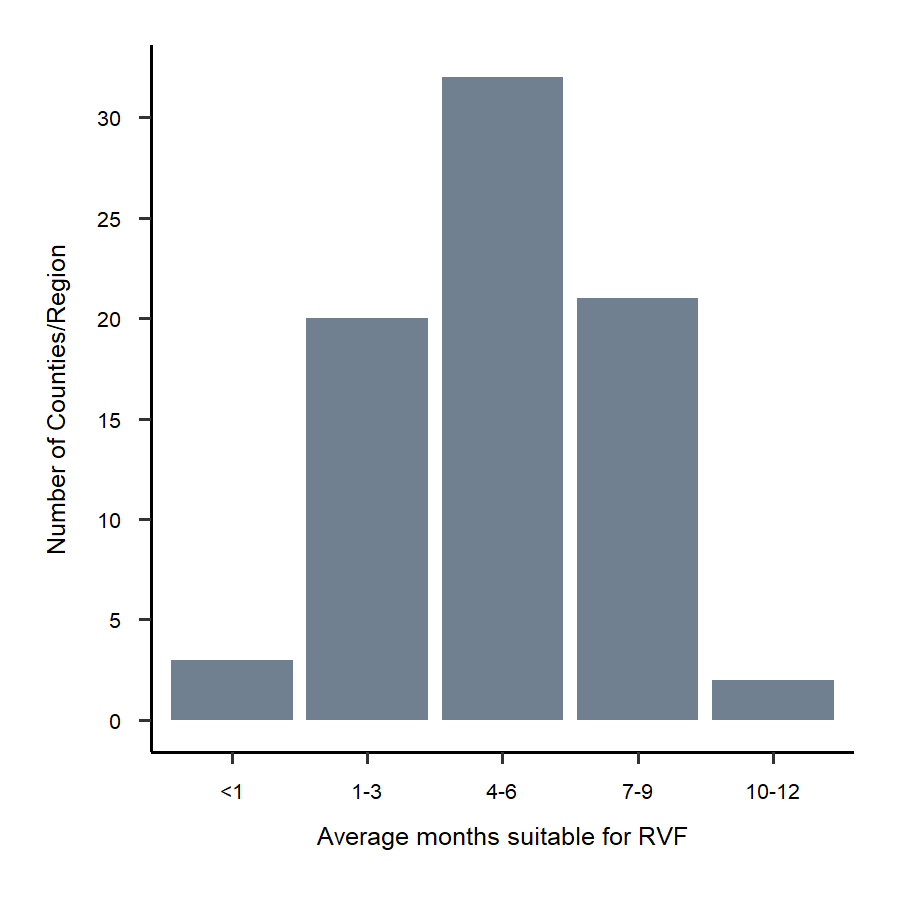
**S3 Figure. The average months where Counties of Kenya and Regions of Tanzania are suitable for RVF.** Using the risk data from Newcastle et al [34] and calculating an average for each county/region based on the sub-county and district level risk data.


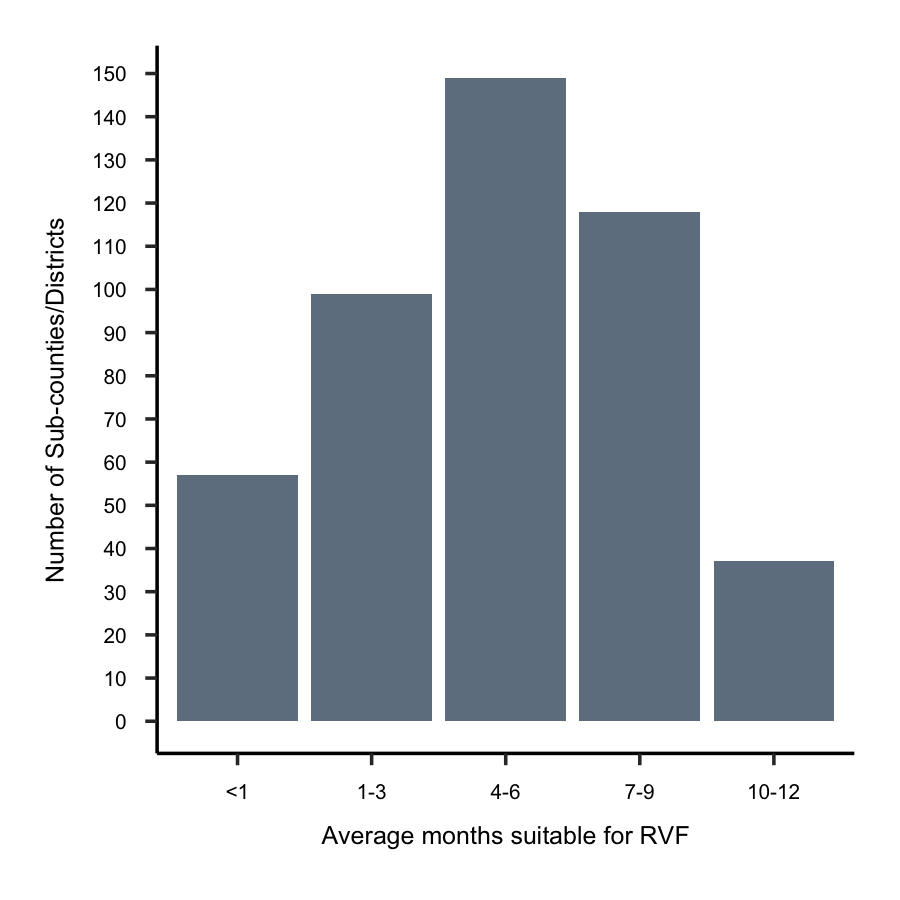
**S4 Figure. The average months where Sub-counties of Kenya and districts of Tanzania are suitable for RVF.** Using the district/sub-county level risk data from Newcastle et al [34].
